# Design and Testing of the Safety Agenda Mobile App for Managing Health Care Managers’ Patient Safety Responsibilities

**DOI:** 10.2196/mhealth.5796

**Published:** 2016-12-08

**Authors:** José Joaquín Mira, Irene Carrillo, Cesar Fernandez, Maria Asuncion Vicente, Mercedes Guilabert

**Affiliations:** ^1^ Alicante-Sant Joan Health District, Consellería Sanitat Alicante Spain; ^2^ Health Psychology Department Miguel Hernández University Elche Spain; ^3^ Systems Engineering and Automation Department Miguel Hernández University Elche Spain

**Keywords:** patient safety, mobile apps, administrators, health service

## Abstract

**Background:**

Adverse events are a reality in clinical practice. Reducing the prevalence of preventable adverse events by stemming their causes requires health managers’ engagement.

**Objective:**

The objective of our study was to develop an app for mobile phones and tablets that would provide managers with an overview of their responsibilities in matters of patient safety and would help them manage interventions that are expected to be carried out throughout the year.

**Methods:**

The Safety Agenda Mobile App (SAMA) was designed based on standardized regulations and reviews of studies about health managers’ roles in patient safety. A total of 7 managers used a beta version of SAMA for 2 months and then they assessed and proposed improvements in its design. Their experience permitted redesigning SAMA, improving functions and navigation. A total of 74 Spanish health managers tried out the revised version of SAMA. After 4 months, their assessment was requested in a voluntary and anonymous manner.

**Results:**

SAMA is an iOS app that includes 37 predefined tasks that are the responsibility of health managers. Health managers can adapt these tasks to their schedule, add new ones, and share them with their team. SAMA menus are structured in 4 main areas: information, registry, task list, and settings. Of the 74 users who tested SAMA, 64 (86%) users provided a positive assessment of SAMA characteristics and utility. Over an 11-month period, 238 users downloaded SAMA. This mobile app has obtained the AppSaludable (HealthyApp) Quality Seal.

**Conclusions:**

SAMA includes a set of activities that are expected to be carried out by health managers in matters of patient safety and contributes toward improving the awareness of their responsibilities in matters of safety.

## Introduction

### Patient Safety

Patients do not expect to be harmed in the course of treatment to recover their health. Professionals also do not expect this result. However, over the course of administering health care, incidents do occur unexpectedly and involuntarily that cause harm to patients (adverse events) [1]. Latent errors within the organization and clinical errors are considered the most common causes of avoidable adverse events [2].

In the Organisation for Economic Co-operation and Development’s member countries, 9% of hospitalized patients [1] suffer an adverse event, whereas studies in primary care centers have identified a rate of adverse events of less than 2% for all consultations [3,4]. At hospitals and primary care centers, 18% and 7% of patients, respectively, experienced more than 1 adverse event [4,5]. Almost half of hospital adverse events were preventable [1]. In developing countries, the prevalence of adverse events in hospitals oscillates around 10.5%, whereas the prevalence of adverse events in ambulatory care is around 5% [6].

The total cost of a lack of patient safety (hospitals, primary care centers, and medication errors in patients) can reach 5.6% [7] of the total health expenditure. In Europe, the cost of adverse events in hospitals has been calculated to vary between 1.5% and 2.4% of the total health expenditure [8,9].

Adverse events in European countries cause 3.5 million disability-adjusted life-years every year, of which 1.5 million are likely due to preventable adverse events. These findings have recently been corroborated (TB Agbabiaka, unpublished data, 2016).

### Responsibility of Managers on Patient Safety

Reducing the frequency of preventable adverse events is a challenge for health care organizations. However, sometime patients suffer an adverse event. When it occurs, healthcare professionals should seek: first, that the same patient does not suffer more than 1 adverse event over the course of treatment; and second, that the same adverse event is not repeated. To achieve this, the health manager’s role is crucial, although the organizations must count on involvement by all their professionals [10]. Root cause analysis, critical incident analysis, and incident simulations are the most useful techniques for investigating what happened [11-13]. A Web-based tool named BACRA (based on root cause analysis, in Spanish Basado en Análisis Causa-RAíz) has been developed to involve frontline health care professionals and middle managers in implementing solutions to prevent recurrence of these incidents [14].

A culture of safety at health care centers is a factor that contributes to reduced risks for patients [15]. However, studies [11] have suggested that many times managers do not spend enough time and do not outline a clear hospital strategy on quality and safety. They do not exert enough effort to shape a positive safety culture, lack awareness of what activities they are expected to do, and do not always ensure an appropriate information system to help clinicians improve their practice [12].

Although health managers have the responsibility of advocating a proactive culture of patient safety and defining a safety framework in health care organizations, there are no available tools to assist with raising the awareness of managers with respect to their responsibilities and roles in patient safety.

The objective of our study was to develop an app for mobile phones and tablets that would provide managers at hospitals and primary care centers with an overview of their responsibilities in matters of patient safety and would help them manage and record the interventions that are expected to be carried out throughout a year in order to properly manage the risks inherent to the health care that patients receive.

## Methods

### SAMA development

**Figure 1:**
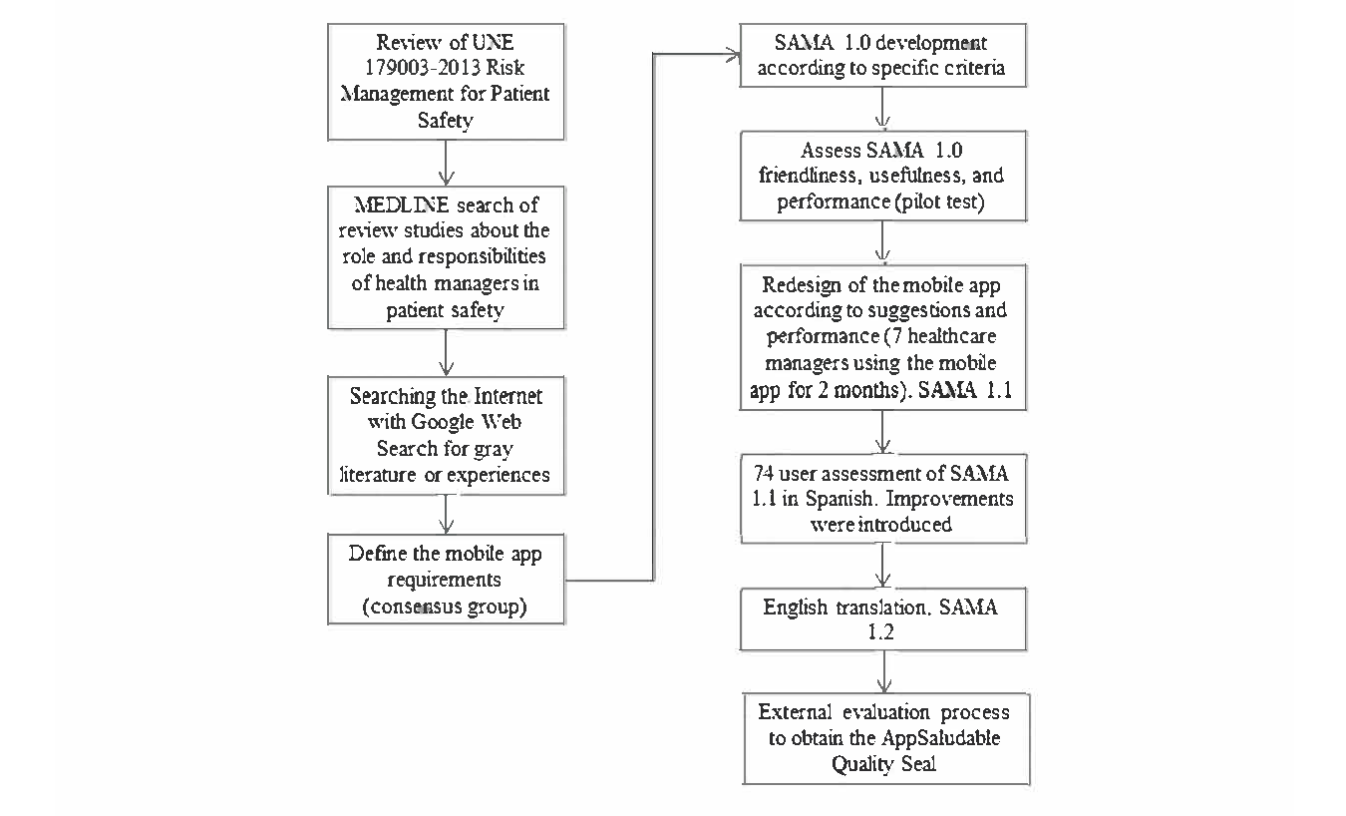
Overview of the steps involved in Safety Agenda Mobile App (SAMA) design and development. UNE: Una Norma Española.

Study design of an app to involve hospital managers and staff on an annual patient safety set of actions. Figure 1 shows the steps followed in the development of the Safety Agenda Mobile App (SAMA).

### Information Sources

A literature review, the Spanish standard Una Norma Española (UNE) 179003:2013 of Risk Management for Patient Safety [13], and a qualitative consensus technique were used to collect information about managers’ responsibilities on patient safety.

The narrative review of the scientific and gray literature identified a set of interventions (activities) on matters of safety that are the responsibility of managers and should be carried out throughout the year. To do this, first a search was conducted using MEDLINE for review studies on the role and responsibilities of managers in patient safety from 2000 to 2015 with a combination of the words *leader* and *managers* with the following descriptors: “patient safety” and “adverse events.” Using the Google meta-search engine, websites that could provide further information on the role and responsibilities of managers with respect to patient safety were analyzed.

This review yielded a selection of studies as information sources. The study by White et al [16] was used to describe the role of managers in patient safety. Rodrigues et al [17] described the reach of the studies and the interventions in patient safety. Research by Clarke et al [18], White et al [19], and Parand et al [11] were the main sources of information for identifying the tasks and responsibilities of managers, along with studies by Van Gerven et al [20] and Mira et al [21] concerning the role of managers in reducing the impact of adverse events on professionals and on the very reputations of health care institutions.

Second, the Spanish standard UNE 179003:2013 of Risk Management for Patient Safety [13] describes a set of appropriate and expected patient safety guidelines that ensure that health care risks are effectively managed. The UNE 179003:2013 forces to deploy operating procedures aimed at reducing the incidence of adverse events from a risk management perspective. This standard was used to elaborate a list of the activities that managers are responsible for was completed.

Third, a consensus group was formed in April 2015 with voluntary participation by 6 patient safety experts. They responded to what characteristics and functions a mobile app is expected to have with the aim of involving managers as leaders on matters of patient safety. The consensus group participants reviewed and prescribed the set of tasks managers are responsible for and also established the requirements that the mobile app should fulfill.

On the basis of all this information, version 1.0 (beta) of the mobile app was designed.

### Assessment of the Safety Agenda Mobile App

To test the operation of SAMA 1.0 and compile ideas about improving this first version, a group of 7 managers from 6 regional health services in Spain were asked to use the app for 2 months. In this initial evaluation, they considered the ease of access and use, the suitability of its contents, the feasibility of the proposed activities, and usefulness of its different functions. This experience permitted redesigning SAMA, and resulted in version 1.1, with improvements implemented in its functions and in the navigation of various menus. This new version of the mobile app, once the improvements were made, was uploaded to the App Store and disseminated in health manager forums within Spain.

In June 2015, SAMA 1.1 became available at the App Store and interested users could download it [22]. This version included 4 nonmandatory questions about utility, strengths, and improvements needed. Four months subsequent to the SAMA launch, the system asked its users to express their opinions about the mobile app; they were also able to propose suggestions for improvement in terms of content, design, and navigation.

SAMA was evaluated externally to assure the appropriateness of the design, quality and safety information provided, confidentiality and privacy, and services provided by the App. This is part of the Quality and Safety Strategy for Spanish health mobile apps. The apps are blind reviewed in a set of 31 criteria before obtaining this AppSaludable (HealthyApp) Quality Seal. The HealthyApp Quality Seal is part of a validation process conducted by an international quality institution in Spain [23].

## Results

### SAMA characteristics

SAMA 1.0 was organized in general content blocks, called activities, and in a set of specific tasks. Table 1 shows the scope of expected SAMA activities. A total of 37 tasks were extracted from the information sources used. They are organized in 8 activities: (1) identification and analysis of risk management processes; (2) analysis of outcomes and risk management monitoring; (3) training actions in patient safety; (4) communication, information, and documentation; (5) consequences of adverse effects; (6) audits; (7) positive safety culture; and (8) management agreements on patient safety aims.

**Table 1:** Activities and subcategories in which the Safety Agenda Mobile App’s tasks are organized (N=37).

Activities and subcategories		Tasks n (%)
**Activity 1. Identification and analysis of risk management processes**		6 (16)
	Risk management processes	1 (17)
	Resources	1 (17)
	Study of adverse event frequency	1 (17)
	Risk analysis	2 (33)
	Clinical sessions	1 (17)
**Activity 2. Analysis of outcomes and risk management at center**		9 (24)
	RCA^a^ outcomes	1 (11)
	Notification: safety committee	2 (22)
	Notification: management committee	2 (22)
	Anonymous notifications	3 (33)
	Effectiveness tracking	1 (11)
**Activity 3. Training actions in patient safety**		6 (16)
	Training revision	3 (50)
	New staff incorporations	2 (33)
	Effectiveness of training actions	1 (17)
**Activity 4. Communication, information, and documentation**		3 (8)
	Communication plan	2 (67)
	Documentation	1 (33)
**Activity 5. Consequences of adverse events**		7 (19)
	Information to relatives	1 (14)
	Victim compensation report	1 (14)
	Insurance policy report	1 (14)
	Impact on second victim	2 (29)
	Impact on third victim	2 (29)
**Activity 6. Audits**		2 (5)
	Revision and planning of the internal and external audit program	2 (100)
**Activity 7. Positive safety culture**		2 (5)
	Foster safety culture within the organization	2 (100)
**Activity 8. Management agreements on patient safety aims**		2 (5)
	Management agreements	2 (100)

^a^RCA: root cause analysis.

The recommended periodicity of most predefined tasks was annual (n=28), but there were some that were recommended to be executed every 3 (n=1), 4 (n=1), or 6 months (n=7).

The requirements to be fulfilled by SAMA were defined by the consensus group and included the following:

- A list of activities that managers at health centers are responsible for, based on the UNE 179003:2013 standard and criteria suggested by scientific documents and expert participants.
- The possibility of personalizing the activities throughout the year, adapting all interventions to each user’s work plan.
- Associating text to each activity as a way to remind, summarize tasks, etc.
- A report of activities generated automatically, which permits monitoring the degree of compliance of the activities for which managers are responsible.
- User-friendly interface. If it is not, users will not continue using the app.
- Highly configurable. Not all hospitals and not all managers follow the same procedures on safety issues.
- High level of privacy. Data entered into the app are extremely sensitive, so they should be kept locally within the device and never uploaded to external servers.
- Access by log-in and password to keep data secure.
- Capability of gathering (anonymous, nonsensitive) data via surveys. Concretely, an initial survey before using the app for the first time and a second survey after several months of use.

### Safety Agenda Mobile App Design and Redesigns

After analyzing the 2 main mobile platforms (Android and iOS), iOS was selected because of various reasons: first, the iPhone is the most common device possessed by prospective app users (management staff); second, fragmentation is much lower in iOS and, thus, it is easier to ensure that the app will work properly on all devices. Besides, the operating system user update rate is higher compared with Android, so there is no need to develop an app capable of working properly on old versions of the operating system. Finally, all iOS apps follow a strict review process by Apple (this is not the case for Android apps). This review process assures app quality indirectly (ie, there are no memory losses, etc).

**Figure 2:**
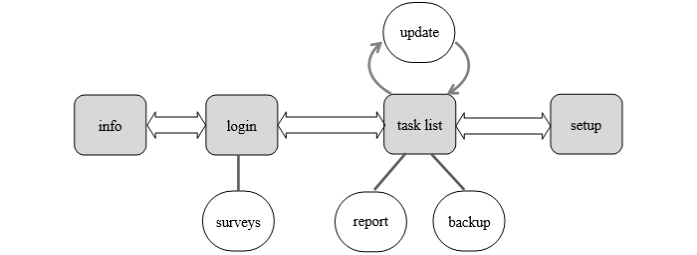
Mobile app structure.

After the pilot tests by initial users (N=7), the following modifications were suggested: first, more customization options, such as the capability of creating new tasks and groups of tasks other than those recommended by the UNE standard and that are shown by default in the app; second, a way for the user to add text notes to certain tasks; third, functionality for backing up data; and, finally, slight changes in usability (mainly for task scheduling). All suggested modifications were carried out, and a second version of the app (version 1.1) was released. SAMA was structured in 4 main areas (see Figure 2):

1. *Info area*, which provides information about the app and the project.

2. *Log-in area*, which is responsible for access control and is also the area where users are asked to fill out surveys.

3. *Task list area*, the main area of the app, which shows the list of tasks ordered by immediacy and allows the user to update such tasks when there are new schedules or when new meetings have been carried out. Besides, the user can generate reports and can also create backup copies from this area.

4. *Setup area*, from where it is possible to customize the app: the user can enable or disable tasks or activities (groups of tasks), and they can also add tasks of their own.

#### Info Area

**Figure 3:**
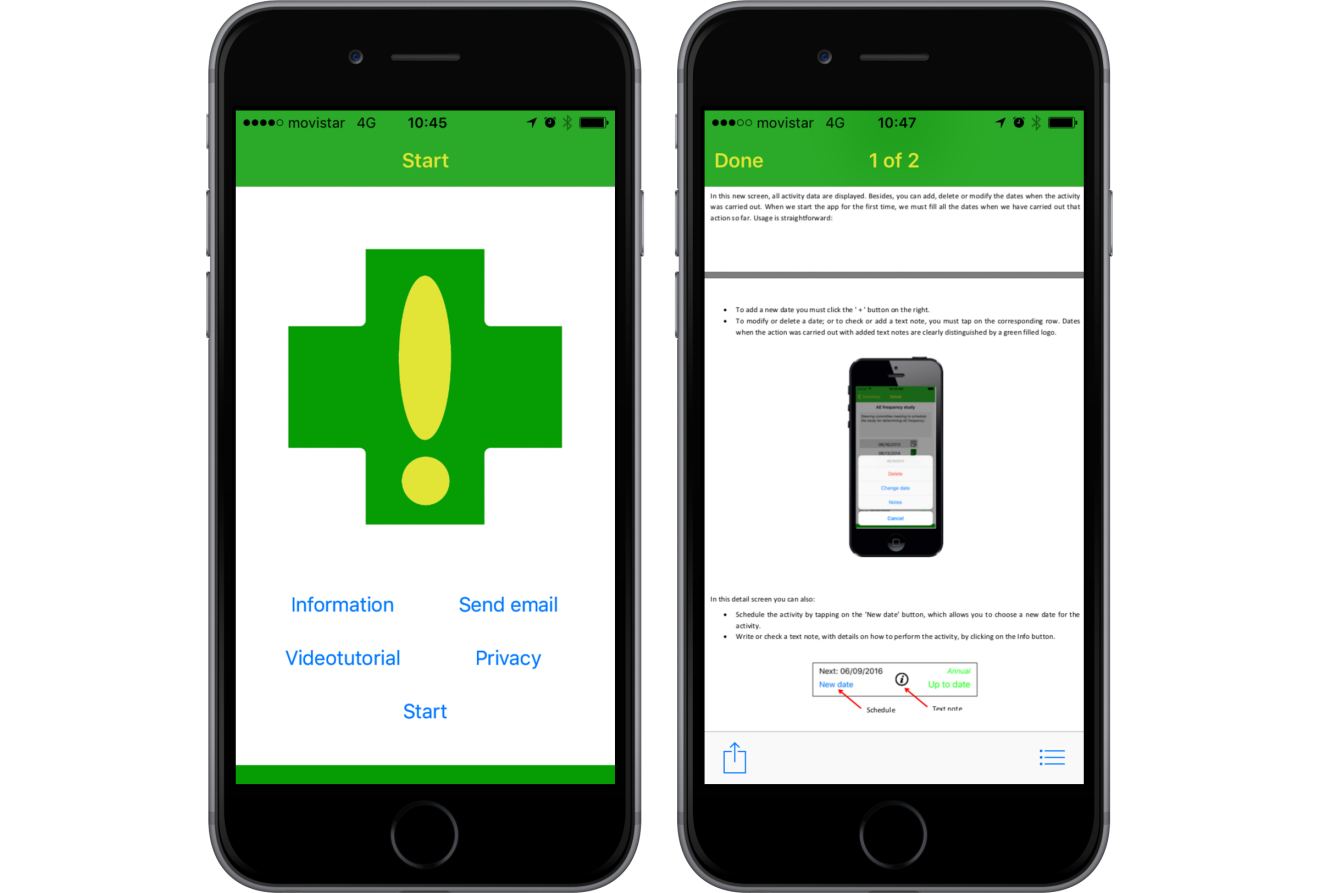
Info area. First screen (left); PDF document with information about the project (right).

The screen that appears when the app is run for the first time is shown in Figure 3 (left). From this screen, the user can access information about the app and the project, both as a PDF document [24] (Figure 3, right) and as a video tutorial [25].

#### Log-In Area

Access to the app is restricted by username and password.

#### Task List Area

**Figure 4:**
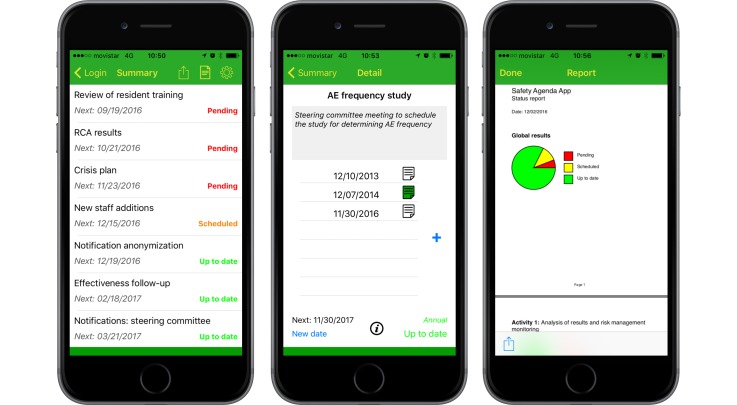
Task list area. Task list (left), detail list (center), and report on status (right).

The main screen in this area shows the list of tasks ordered by immediacy. Every task has a certain periodicity, so it has to be carried out at regular times. There are 3 possible states for a task: pending (ie, its due date has arrived and it has not been completed or scheduled), scheduled (ie, its due date has been postponed by the user), and up-to-date (ie, its due date has not yet arrived). Figure 4 (left) shows this screen.

Tapping one of the tasks takes you to a detailed screen where you can update the task status: you can schedule the task for a later date, you can mark this task as completed on a certain date, and you can also add additional text information. Figure 4 (center) shows the detail screen.

Additionally, there are 2 more actions that can be performed from the task list screen: you can generate a PDF report on the status of all tasks, like Figure 4 (right) shows, and you can also create a backup copy of your data.

#### Setup Area

**Figure 5:**
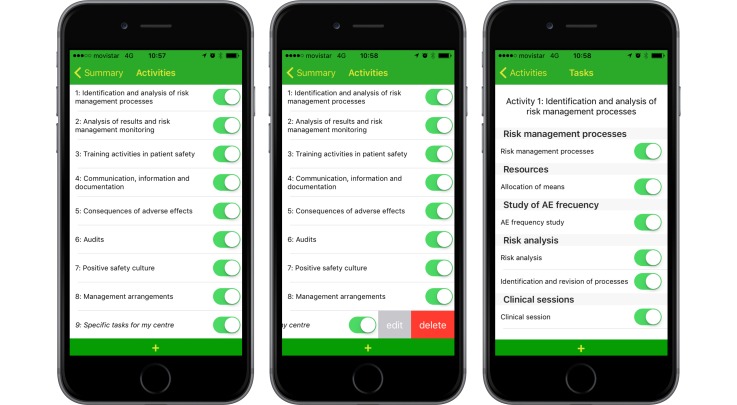
Setup area. Activity list (left), deleting or editing your own activities (center), and detail screen with the task list for a certain activity (right).

App customizations are performed in the setup area. Basically, users can enable or disable tasks depending on users’ hospital or primary care needs and also create their own custom tasks. Tasks are grouped into activities and sections. Figure 5 (left) shows the first setup screen, where users can enable or disable activities (groups of tasks) and create their own activities. Figure 5 (center) shows that you can edit these activities just by swiping the row to the left. Finally, Figure 5 (right) shows the second setup screen, or detail view, where you can enable or disable each task independently; you can also create your own tasks.

Once the user has been identified, the list of tasks included in the agenda appears along with their status, which can be pending (when the task has yet to be done within the recommended implementation period), up-to-date (when the date of a finished task is introduced that is within the recommended implementation period), and programmed (by introducing an upcoming date to finish a task that was either pending or up-to-date). Each task is previously introduced into the system with a recommend schedule for completion (four times a year, three times, twice, or once a year). Touching any specific task makes its description and its compliance record appear, and the system permits users to add the last date that the task was completed, thereby modifying its status. The app has an edit screen that permits users to either hide or show specific tasks or groups of tasks (activities). SAMA permits downloading and emailing of compliance records.

### Safety Agenda Mobile App 1.1 Assessment

Between June and September 2015, a total of 102 users downloaded the mobile app, and of these, 74 (72%) registered and assessed SAMA 1.1. Among those registered, it was more frequent that the management team of the health institution analyzed the risks to patient safety and shared its conclusions with the quality and safety commissions, and instructors (Table 2). As for its main strengths, these users identified that SAMA 1.1 constitutes a reminding system of the responsibilities of patient safety managers at the same time as it emerged as a verification list of safety tasks. The aspects needing improvement, according to user opinions, had to do with its design (user-friendliness, background color, font size) and with additional functions such as personalizing the content of the compliance reports, prioritizing tasks, synchronizing them with calendar applications, and expanding the task status scale (Table 3).

Version 1.2, which is about to be released, supports the English language (versions 1.0 and 1.1 supported only Spanish). SAMA 1.2 is also available at the App Store. A total of 238 users downloaded the mobile app till June 2016, either the version in Spanish, SAMA 1.1, or the version in English, SAMA 1.2.

As the current version of the app (version 1.1) only supports the Spanish language, most downloads correspond to Spain (167/238, 70.2%). However, the number of downloads in other countries suggest that version 1.2 (which supports the English language and is about to be released) will be accepted well worldwide (71/238, 29.8%).

On November 24, 2016, SAMA has been accredited receiving the AppSaludable (HealthyApp) Quality Seal [26]. This mean that SAMA meets a set of criteria about design, functionality, safety and quality and could be used in the health context to provide managers with an overview of their responsibilities in matters of patient safety.

**Table 2:** Focus and management activity in patient safety (N=74).

Users experiences (quantitative data)	Yes (%)	No (%)
The most frequent causes of adverse events are periodically reviewed with the steering committee.	54 (73)	20 (27)
The management team dedicates at least one session every 4 months to analyze the risks to patient safety and shares the conclusions with the quality and safety commissions, and instructors.	41 (55)	33 (45)
SAMA^a^ 1.1 assessed positively on its usefulness and navigation facilities	64 (87)	10 (13)

^a^SAMA: Safety Agenda Mobile App.

**Table 3:** Safety Agenda Mobile App 1.1 strengths and areas for improvement identified by users (N=74).

Users experiences (qualitative data)		n
**Strengths**		
	Reminds managers about tasks	5
	Facilitates a checklist of safety tasks	3
	Clear action scheme	2
	Easy to use	2
	Very intuitive	1
**Areas for improvement**		
	Trial design	1
	Small font size	1
	Wallpaper color cannot be chosen	1
	Ability to print the report without the graphic summaries	1
	Ability to classify items to comply with prioritizing the tasks	1
	Ability to transfer dates to Outlook or other calendar schedules	1
	It could consider other degrees of the status of each task (eg, not started, started, in progress, final phase, finished, halted)	1

## Discussion

### Principal Findings

SAMA was designed for the managing team at a health center to become aware of all the activities in matters of patient safety that are their responsibility and to facilitate their involvement directing these activities that are planned throughout the year. The agenda’s format allows programming the tasks in a manner consistent with the center’s patient safety plan and personalizing the tasks according to such plan and the annual forecasts. Moreover, SAMA permits sharing activities with the center’s middle management and editing a report of the activities carried out that reflect the degree of commitment by the management to patient safety. SAMA includes a set of activities that are expected to be carried out by health managers in matters of patient safety and contributes toward improving the awareness of their responsibilities in matters of safety.

### SAMA Utility

The review by Parand et al [11] showed that when managers incorporate quality and safety objectives into their work agendas, health care center results improve. SAMA adopts these results as a premise and goes further, incorporating the responsibilities in matters of patient safety that the UNE 179003:2013 standard incorporates in the management of risks inherent in health intervention into the design and promotion of a culture of safety at the center, to the review of outcome indicators, etc.

Not having a vision of the tasks that must be performed on matters of safety is one of the main problems for managers—for this, SAMA provides a dynamic, flexible, and trustworthy structure that helps management staff conceptualize their responsibilities in matters of safety and include them in their work agendas. Another problem is not having sufficient time for all the tasks that are expected to be carried out throughout a year in safety matters. Time management, the delegation of tasks, and appropriate input of all the activities expected of managers in matters of safety are the ways in which SAMA helps them overcome the identified gaps.

Progressively, we count on evidence of the effectiveness of the proposed interventions to reduce the occurrence and impact of adverse events. The projects of Bacteremia Zero (in Spain, it achieved in 1 year a reduction of some 3800 cases of bacteremia associated with central venous catheter in the intensive care unit or ICU [27]) and Pneumonia Zero (a reduction of pneumonia associated with mechanical ventilation in ICUs to 6 per 1000 mechanical ventilation days in 1 year [28]) are just 2 examples. These actions cannot be an option; instead, the responsibility of managers is to do whatever possible to incorporate them in their centers. SAMA is designed to be customized and permits introducing those actions that are considered effective and necessary.

### Conclusion

The culture of safety of health care organizations has been related with the outcome of the risk management that affects the number of adverse events patients suffer [29]. Regrettably, a people approach is still preferred over a system approach, and we forget that a safe system is that which reduces the probability of professionals committing errors [30]. SAMA, just like other tools such as those designed for reporting systems [31], can contribute to implementing a positive and solid culture of safety in this case because it facilitates a set of activities managers are responsible for in order to accomplish proper management of the risks inherent to health care.

### Limitations

The current SAMA design is limited to use by only iOS devices. The effect SAMA may have on the center’s culture of safety has not been analyzed, nor whether its use affects the risk management for patient safety to any extent.

### Future Studies

This study requires future research to determine the impact on patient safety indicators resulting from continuous use of SAMA at health care centers. New app updates could incorporate modifications based on user comments, and a version for Android operating systems could even be built.

## Abbreviations

ICU: intensive care unit
SAMA: Safety Agenda Mobile App
UNE: Una Norma Española

## References

[1] de Vries EN, Ramrattan MA, Smorenburg SM, Gouma DJ, Boermeester MA (2008). The incidence and nature of in-hospital adverse events: a systematic review. Qual Saf Health Care.

[2] Seys D, Wu AW, Van Gerven E, Vleugels A, Euwema M, Panella M, Scott SD, Conway J, Sermeus W, Vanhaecht K (2013). Health care professionals as second victims after adverse events: a systematic review. Eval Health Prof.

[3] Tsang C, Bottle A, Majeed A, Aylin P (2013). Adverse events recorded in English primary care: observational study using the General Practice Research Database. Br J Gen Pract.

[4] Aranaz-Andrés JM, Aibar C, Limón R, Mira JJ, Vitaller J, Agra Y, Terol E (2012). A study of the prevalence of adverse events in primary healthcare in Spain. Eur J Public Health.

[5] Allué Natalia, Chiarello Pietro, Bernal Delgado Enrique, Castells Xavier, Giraldo Priscila, Martínez Natalia, Sarsanedas Eugenia, Cots Francesc (2014). [Assessing the economic impact of adverse events in Spanish hospitals by using administrative data]. Gac Sanit.

[6] Montserrat-Capella D, Suárez M, Ortiz L, Mira JJ, Duarte HG, Reveiz L (2015). Frequency of ambulatory care adverse events in Latin American countries: the AMBEAS/PAHO cohort study. Int J Qual Health Care.

[7] Antoñanzas VF (2013). [Non safety costs in the Spanish health care system]. Rev Esp Salud Publica.

[8] Hoonhout LH, de Bruijne MC, Wagner C, Zegers M, Waaijman R, Spreeuwenberg P, Asscheman H, van der Wal G, van Tulder MW (2009). Direct medical costs of adverse events in Dutch hospitals. BMC Health Serv Res.

[9] Allué N, Chiarello P, Bernal DE, Castells X, Giraldo P, Martínez N, Sarsanedas E, Cots F (2014). [Assessing the economic impact of adverse events in Spanish hospitals by using administrative data]. Gac Sanit.

[10] Etchegaray JM, Thomas EJ (2015). Engaging Employees: The Importance of High-Performance Work Systems for Patient Safety. J Patient Saf.

[11] Parand A, Dopson S, Renz A, Vincent C (2014). The role of hospital managers in quality and patient safety: a systematic review. BMJ Open.

[12] Mira JJ, Carrillo I, Lorenzo S, Ferrús L, Silvestre C, Pérez-Pérez P, Olivera G, Iglesias F, Zavala E, Maderuelo-Fernández JÁ, Vitaller J, Nuño-Solinís R, Astier P, Research Group on Second and Third Victims (2015). The aftermath of adverse events in Spanish primary care and hospital health professionals. BMC Health Serv Res.

[13] (2013). AENOR.

[14] Carrillo Irene, Mira José Joaquín, Vicente Maria Asuncion, Fernandez Cesar, Guilabert Mercedes, Ferrús Lena, Zavala Elena, Silvestre Carmen, Pérez-Pérez Pastora (2016). Design and Testing of BACRA, a Web-Based Tool for Middle Managers at Health Care Facilities to Lead the Search for Solutions to Patient Safety Incidents. J Med Internet Res.

[15] McFadden Kathleen L, Stock Gregory N, Gowen Charles R (2015). Leadership, safety climate, and continuous quality improvement: impact on process quality and patient safety. Health Care Manage Rev.

[16] White AA, Brock DM, McCotter PI, Hofeldt R, Edrees HH, Wu AW, Shannon S, Gallagher TH (2015). Risk managers' descriptions of programs to support second victims after adverse events. J Healthc Risk Manag.

[17] Rodrigues SP, van Eck NJ, Waltman L, Jansen FW (2014). Mapping patient safety: a large-scale literature review using bibliometric visualisation techniques. BMJ Open.

[18] Clarke JR, Lerner JC, Marella W (2007). The role for leaders of health care organizations in patient safety. Am J Med Qual.

[19] White AA, Waterman AD, McCotter P, Boyle DJ, Gallagher TH (2008). Supporting health care workers after medical error: considerations for healthcare leaders. J Clin Outcomes Manag.

[20] Gerven EV, Seys D, Panella M, Sermeus W, Euwema M, Federico F, Kenney L, Vanhaecht K (2014). Involvement of health-care professionals in an adverse event: the role of management in supporting their workforce. Pol Arch Med Wewn.

[21] Mira JJ, Lorenzo S, Carrillo I, Ferrús L, Pérez-Pérez P, Iglesias F, Silvestre C, Olivera G, Zavala E, Nuño-Solinís R, Maderuelo-Fernández JÁ, Vitaller J, Astier P, Research Group on Second and Third Victims (2015). Interventions in health organisations to reduce the impact of adverse events in second and third victims. BMC Health Serv Res.

[22] Appandabout SL https://itunes.apple.com/es/app/safety-agenda-mobile-app/id1008897674?l=en&mt=8.

[23] Calidad.

[24] Mira JJ, Carrillo I, Fernández C, Vicente MA, Guilabert M http://appandabout.es/sama/info_safetyagenda.pdf.

[25] Mira JJ, Carrillo I, Fernández C, Vicente MA, Guilabert M https://www.youtube.com/watch?v=d5-8NacXwZw.

[26] Andalusian Agency for Health Care Quality http://www.calidadappsalud.com/distintivo/info?app=safety-agenda-mobile-sama.

[27] Palomar M, Álvarez-Lerma F, Riera A, Díaz MT, Torres F, Agra Y, Larizgoitia I, Goeschel CA, Pronovost PJ, Bacteremia Zero Working Group (2013). Impact of a national multimodal intervention to prevent catheter-related bloodstream infection in the ICU: the Spanish experience. Crit Care Med.

[28] Álvarez-Lerma F, Álvarez J, Añón JM, Arias S, García R, Gordo F, Jam R, Lorentz L, Palomar M, Sánchez M (2014). Informe final del proyecto Neumonía Zero. En: X Congreso Panamericano e Ibérico de Medicina Crítica y Terapia Intensiva. Med Intensiva.

[29] Wakefield JG, McLaws M, Whitby M, Patton L (2010). Patient safety culture: factors that influence clinician involvement in patient safety behaviours. Qual Saf Health Care.

[30] Panella M, Leigheb F, Rinaldi C, Donnarumma C, Tozzi Q, Di Stanislao F (2015). [Defensive Medicine: Defensive Medicine: Overview of the literature]. Ig Sanita Pubbl.

[31] Elliott P, Martin D, Neville D (2014). Electronic clinical safety reporting system: a benefits evaluation. JMIR Med Inform.

